# A draft genome assembly of spotted hyena, *Crocuta crocuta*

**DOI:** 10.1038/s41597-020-0468-9

**Published:** 2020-04-28

**Authors:** Chentao Yang, Fang Li, Zijun Xiong, Klaus-Peter Koepfli, Oliver Ryder, Polina Perelman, Qiye Li, Guojie Zhang

**Affiliations:** 1 BGI-Shenzhen, Shenzhen, 518083 China; 2 China National GeneBank, BGI-Shenzhen, Shenzhen, 518120 China; 3 Section for Ecology and Evolution, Department of Biology, University of Copenhagen, DK-2100 Copenhagen, Denmark; 4 State Key Laboratory of Genetic Resources and Evolution, Kunming Institute of Zoology, Chinese Academy of Sciences (CAS), Kunming, Yunnan 650223 China; 5 Smithsonian Conservation Biology Institute, Center for Species Survival, National Zoological Park, Front Royal, Virginia 22630 and, Washington, DC 20008 USA; 6 Theodosius Dobzhansky Center for Genome Bioinformatics, Saint Petersburg State, University, St. Petersburg, 199034 Russia; 7 San Diego Zoo Institute for Conservation Research, Escondido, CA 92027 USA; 8 Institute of Molecular and Cellular Biology, Lavrentiev ave. 8/2, Novosibirsk, 630090 Russia; 9 Novosibirsk State University, Novosibirsk, 630090 Russia

**Keywords:** Genome, Genome informatics

## Abstract

The spotted hyena (*Crocuta crocuta*), one of the largest terrestrial predators native to sub-Saharan Africa, is well known for its matriarchal social system and large-sized social group in which larger females dominate smaller males. Spotted hyenas are highly adaptable predators as they both actively hunt prey and scavenge kills by other predators, and possess an enhanced hypercarnivorous dentition that allows them to crack open bones and thereby feed on nearly all parts of a carcass. Here, we present a high-quality genome assembly of *C. crocuta* that was generated using a hybrid assembly strategy with Illumina multi-size libraries. A genome of about 2.3 Gb was generated with a scaffold N50 length of 7.2 Mb. More than 35.28% genome region was identified as repetitive elements, and 22,747 protein-coding genes were identified in the genome, with 97.45% of these annotated by databases. This high-quality genome will provide an opportunity to gain insight into the evolution of social behavior and social cognition in mammals, as well as for population genetics and metagenomics studies.

## Abstract (summary)

**Table:** 

Measurement(s)	DNA • genome • sequence_assembly • sequence feature annotation
Technology Type(s)	DNA sequencing • sequence assembly process • sequence annotation
Sample Characteristic - Organism	Crocuta crocuta

Machine-accessible metadata file describing the reported data: 10.6084/m9.figshare.12091458

## Background & Summary

**Fig. 1:**
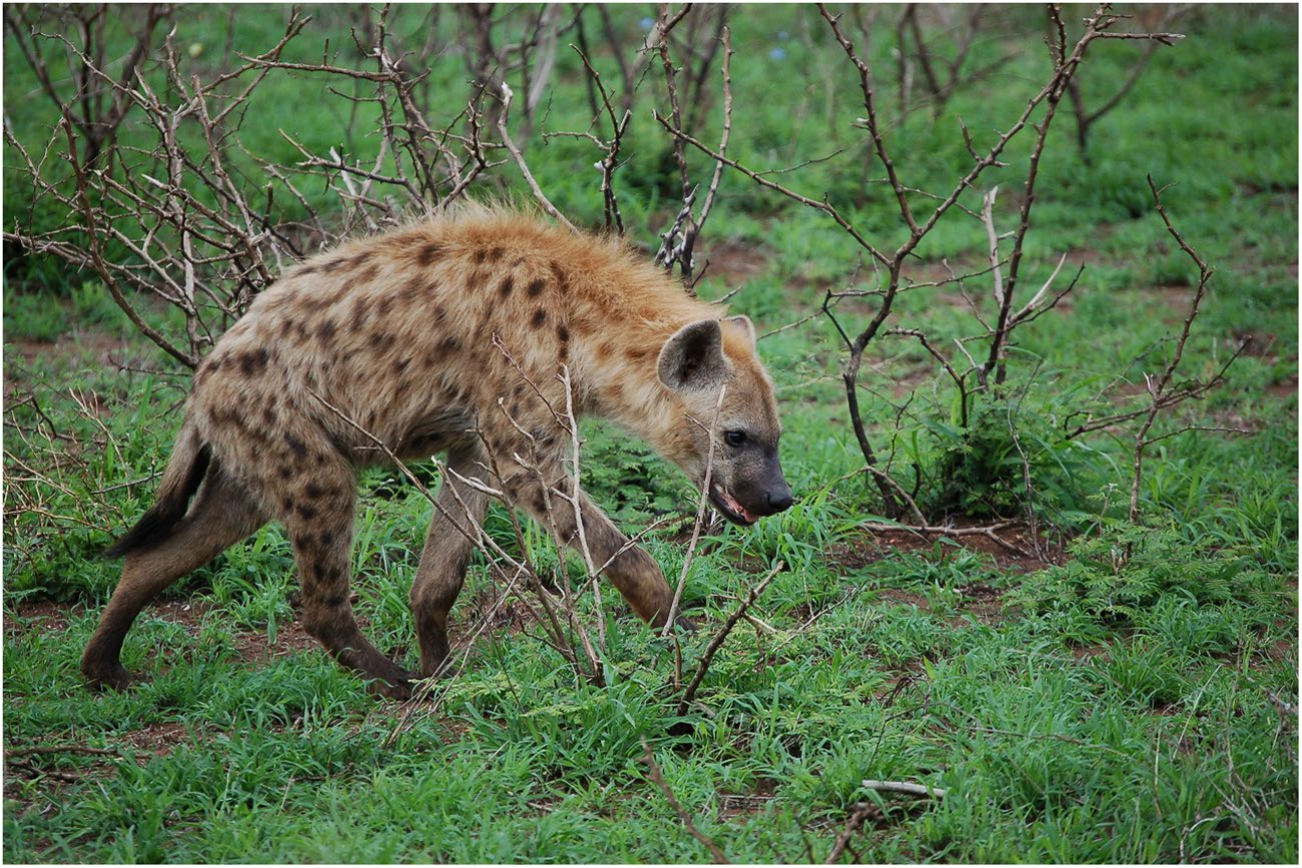
A photograph of an adult *C. Crocuta*. The user *“*garywalker” uploaded this image to https://pixabay.com.

Hyenas (also spelled “hyaena” in some parts of the world; Fig. 1) are among the most common large carnivores in Africa, with a widespread distribution occupying most of the habitats of the continent. There are four living species of hyena - spotted hyena (*Crocuta crocuta*), striped hyena (*Hyaena hyaena*), brown hyena (*Hyaena brunnea*), and aardwolf (*Proteles cristata*). A previous molecular systematics study suggested that hyaenids diverged from their feliform sister group 29.2 MYA, in the Middle Oligocene^1^. The spotted hyena is the largest member of this family and is known for its laughing call. They are fairly large in build, with body weights up to 64 kg and 55 kg for females and males, respectively^2^, and have relatively short torsos with lower hindquarters, and sloping backs. They have excellent night-time hearing and vision and can be found in all habitats except central Afrotropical forests, including savannas, grasslands, woodlands, forest edges, subdeserts, and even mountains up to 4,000 meters^2^.

The spotted hyena displays a number of unusual features that are unique among mammals. As the most numerous large predators, their prey mostly comes from ungulates, such as wildebeest, zebra, Thomson’s gazelles, cape buffalo, impala, and they also feed on insects and fishes^2^. Spotted hyenas have an exceptionally robust dentition, and they have the largest premolars compared with any living carnivora species of the same body size^3^. Adult spotted hyenas can generate powerful bite forces that are associated with their ability to capture prey with body sizes up three times larger than themselves and crush bones using their teeth^4^. These abilities are related to a unique caudally elongated frontal sinus in spotted hyenas that dissipate bending stresses during bone-cracking^5^. Unlike other carnivores, spotted hyenas are not only able to splinter the bones of large ungulates, but they are also able to digest them completely, including all organic components^2^.

However, perhaps the most peculiar feature of spotted hyenas is related to their reproductive biology, which in turn is directly related to their social behavior. Female spotted hyenas are about 10% larger than males and are much more aggressive, resulting in a social system where the masculinized females are dominant to all adult immigrant males^6^. Furthermore, females have evolved a pseudophallus as a result of a greatly elongated clitoris, the formation of which is independent of androgen hormones but may be related to estrogen signaling^7^. However, the behavioral aggressiveness of female hyenas and that displayed between cubs soon after parturition to establish dominance may be mediated by unusually high concentrations of androgens^8^. Therefore, the spotted hyena is a fascinating model species for studying the social behavior, evolution of sexual dimorphism, demography and genetic structure of a gregarious mammalian carnivore. These large predators live in societies that are far larger and more complex than those of any other mammalian carnivore and current studies of spotted hyenas are focused on the social intelligence of hyena societies^9^. Deciphering the genetic underpinnings of these remarkable traits would be greatly facilitated by the generation of a reference genome for spotted hyenas.

The four extant hyaenid species have a conserved karyotype of 2n = 40, with slight differences in the fundamental number of chromosomal arms. The hyaenid karyotype differs from the ancestral Carnivora karyotype by 4 fusions, 3 fissions, and at least 3 inversions as shown by comparative chromosome painting. As with the majority of autosomes, the X chromosome has a large C-positive centromeric region. G-banding patterns of the spotted hyena are very similar to those of the striped hyena^10^. To date, only the genomes of two striped hyenas (a female and a sample without sex information) have been sequenced and assembled^11^ (Genbank accession GCA_003009895.1 and GCA_004023945.1, respectively). The complete mitochondrial genomes have been generated for all four hyena species^11,12^. Here we present the first draft genome of a male spotted hyena (*Crocuta crocuta)*, which will offer opportunities for unraveling the evolutionary history, population genetics and genetic underpinnings of the unique biological features of this endlessly fascinating species.

## Methods

### Sample collection, library construction and sequencing

Genomic DNA was obtained from a male specimen of *C. crocuta* (NCBI taxonomy ID: 9678; Fig. 1) stored in the Frozen Zoo^®^ at the San Diego Zoo Institute for Conservation Research, USA (Frozen Zoo ID: KB4526).

The genomic DNA was extracted using phenol-chloroform followed by purification using ethanol precipitation^13^. The extracted DNA was run and visualized on a 1.5% agarose gel run in 1x TBE buffer to check for the presence of high molecular weight DNA. DNA concentration and purity were quantified on a NanoDrop 2000 spectrophotometer and Qubit 2.0 Fluorometer (Thermo Fisher Scientific, USA) before shipping to BGI-Shenzhen, China. We obtained a total of 372 µg of genomic DNA, with a concentration of 0.418 µg/µL using the Nanodrop 2000 and 0.245–0.399 µg/µL based on four replicate readings using the Qubit 2.0 Fluorometer. The 260/280 ratio of purity was 1.95. We then barcoded the sample using *cytochrome b* (Cytb) gene. Then, according to the gradient library strategy, we constructed 13 insert-size libraries, with the following insert size lengths: 170 bp, 500 bp, 800 bp, 2 kbp, 5 kbp, 10 kbp, 20 kbp. We used the HiSeq. 2000 sequencer (Illumina, USA) to sequence Paired-End (PE) reads for each library across 14 lanes. A total of about 299 Gb raw data was generated from 13 libraries, achieving a sequencing depth (coverage) of 149.25 (Table 1).

**Table 1:** Statistics of raw read data, assuming the genome size is 2.0 Gb.

Insert Size	Library number	Total Data(G)	Reads Length	Sequence coverage (X)
170 bp	2	69.4	100	34.7
500 bp	2	48.6	100	24.3
800 bp	1	42.9	150	21.45
2 kb	2	37.5	49	18.75
5 kb	2	34.3	49	17.15
10 kb	2	35.5	49	17.75
20 kb	2	30.3	49	15.15
Total	13	298.5	—	149.25

### Quality control

To minimize misassembly errors, we filtered raw reads prior to *de novo* genome assembly according to the following two criteria. First, reads with more than 10 bp aligned to the adapter sequence (allowing <= 3 bp mismatch) were removed. Second, reads with 40% of bases having a quality value less than or equal to 10 were discarded. Finally, we obtained 190.4 G data with a coverage of 95.2 (Table 2).

**Table 2:** Data statistics following filtering of raw read data.

Insert Size	Total Data(G)	Reads Length	Sequence coverage (X)
170 bp	63.5	100	31.75
500 bp	44.0	100	22
800 bp	29.7	150	14.85
2 kb	24.1	49	12.05
5 kb	18.3	49	9.15
10 kb	7.1	49	3.55
20 kb	3.5	49	1.75
Total	190.4	—	95.2

### Estimation of genome size

**Fig. 2:**
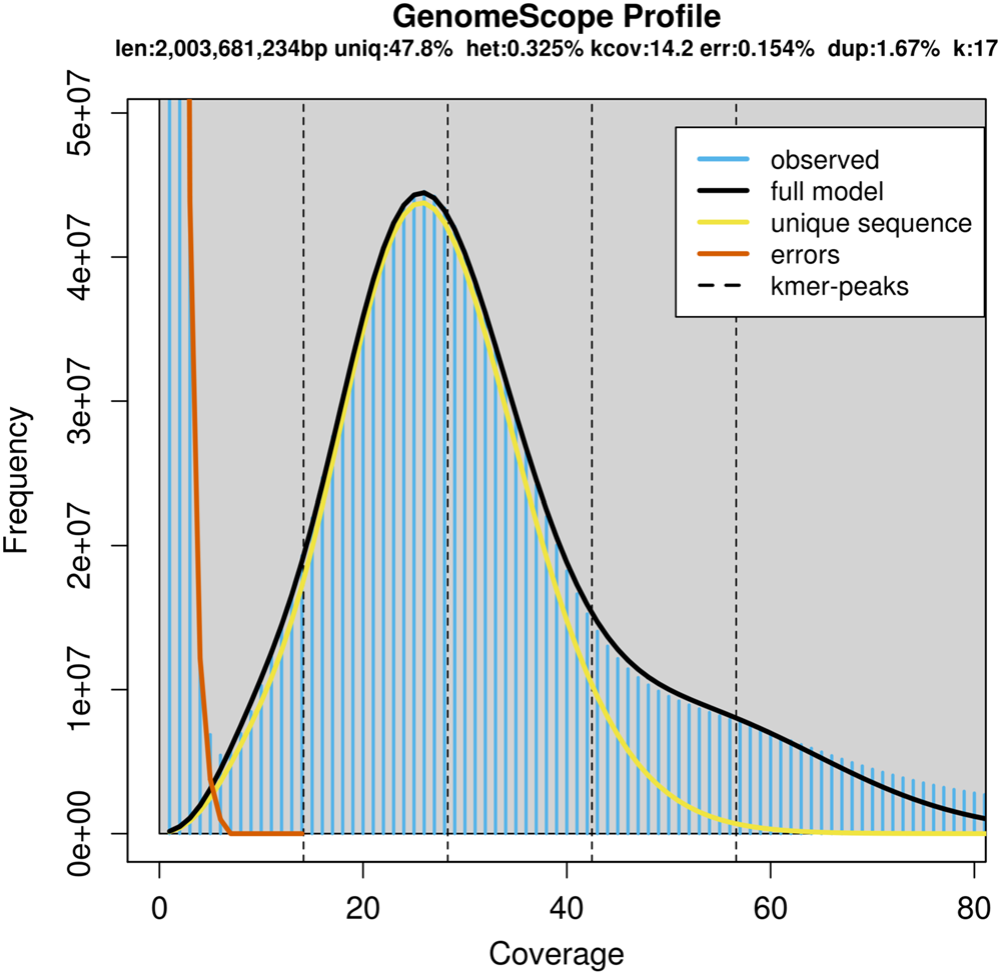
17-mer estimate of genome size. The x-axis is depth (X), the y-axis is the proportion which represents the frequency at that depth divided by the total frequency of all coverage depths. Without consideration of the sequence error rate, heterozygosity rate, and repeat rate of the genome, the 17-mer distribution should approximate a Poisson distribution.

Three short-insert libraries (two of 170 bp and one of 500 bp) were used to estimate the genome size and genome-wide heterozygosity by k-mer analysis. A total of about 385 M PE reads were submitted to jellyfish^14^ to calculate k-mer frequency. Then the k-mer distribution was illustrated by Genomescope^7^ with parameters “k = 17; length = 100; max coverage = 1000”. We obtained an estimated genome size 2,003,681,234 bp, and heterozygosity of 0.325% (Fig. 2).

### Genome assembly and assessment

SOAPdenovo (V1.06)^15^ was employed to assemble the genome de novo, following the filtering of the short insert size data and removing the small peak of the large insert size data. The SOAPdenovo assembly algorithm included three main steps. (1) Contig construction: the short-insert size library data were split into k-mers and constructed using a de *Bruijn* graph, which was simplified by removing tips, merging bubbles, removing the low coverage of the connection and removing small repeats. We obtained the contig sequence by connecting the k-mer path, resulting in a contig N50 2,104 bp, and total length 2,295,545,898 bp. (2) Scaffold construction: we obtained 80% of all aligned paired-end reads by realigning all usable read on contigs. Then we calculated the amount of shared paired-end relationships between each pair of contigs, weighted the rate of consistent and conflicting paired-ends, and then constructed the scaffolds step by step. As a result, we obtained scaffolds with an N50 7,168,038 bp, and total length 2,355,303,269 bp from short insert-sized paired-ends, to long distant paired-ends. (3) Gap closing: To fill the gaps inside the constructed scaffolds, we used the paired-end information to retrieve the read pairs to do a local assembly again for these collected reads. In summary, we closed 87.7% of the intra-scaffold gaps, or 85.8% of the sum gap length. The contig N50 size increased from 2,104 bp to 21,301 bp (Table 3). The scaffold assembly size was 2,355,303,269 bp, which is close to the assembly-based genome size of 2,374,716,107 bp reported for the striped hyaena, *Hyaena hyaena*^11^ (NCBI accession: ASM300989v1). We also retrieved and annotated the mitochondrial genome of the spotted hyena using the MitoZ program^16^, which has a length of 16,858 bp, similar to the first mitochondrial genomes sequenced for this species^12^.

**Table 3:** Statistics of the assembled sequence length.

	Contig		Scaffold	
	Size(bp)	Number	Size(bp)	Number
N90	4,365	121,463	1,189,330	387
N80	8,240	83,172	2,627,146	258
N70	12,224	60,042	4,407,258	190
N60	16,565	43,673	5,630,385	143
N50	21,301	31,246	7,168,038	106
Longest	198,209		23,938,478	
Total Size	2,333,667,234		2,355,303,269	
Total Number (>100 bp)	428,233		171,240	
Total Number (>2 kb)	164,847		2,475	

Note: The above statistics were based on original assemblies, not consistent with the version submitted to NCBI because the sequences shorter than 200 bp were removed before submission.

Assessment of the draft genome was performed by looking at the completeness of single-copy orthologs using BUSCO (version 3.1.0)^17^, searching against Mammaliaodb9 database which contains 4,104 single-copy ortholog groups. A total of 95.5% of the orthologs were identified as complete, 2.5% as fragmented and 2.0% as missing, indicating an overall high quality of the spotted hyena genome assembly. Given that 99.95% of the short scaffolds (<1k) harbored only 1.2% of the total genome length, we excluded these scaffolds for downstream analysis, including repetitive element and gene feature annotation.

### Repetitive element annotation

**Fig. 3:**
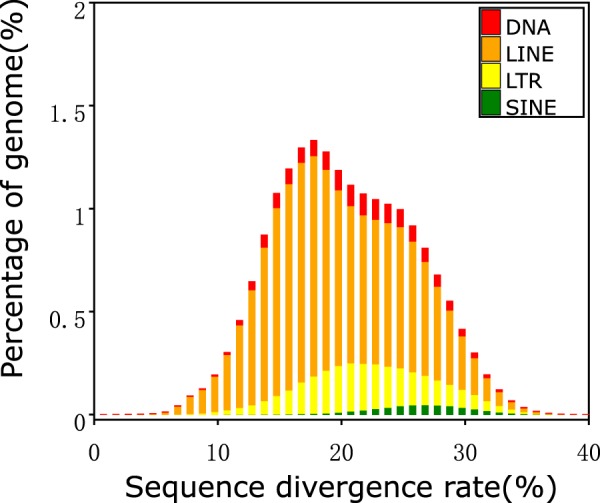
Distribution of divergence rate of each type of transposable element (TE) in the *Crocuta crocuta* genome assembly based on homology-based prediction. The divergence rate was calculated between the identified TEs in the genome using a homology-based method and the consensus sequence in the Repbase database^20^.

**Fig. 4:**
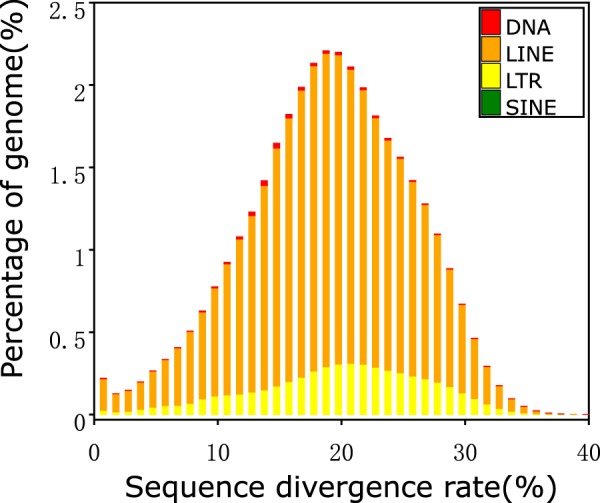
Distribution of divergence rate of each type of TE in the *Crocuta crocuta* genome assembly based on *ab initio* prediction. The divergence rate was calculated between the identified TEs in the genome by *ab initio* prediction and the consensus sequence in the predicted TE library (see Methods).

Both tandem repeats and transposable elements (TE) were searched for and identified across the *C. crocuta* genome. Tandem repeats were identified using Tandem Repeats Finder (TRF, v4.07)^18^ and transposable elements (TEs) were identified by a combination of homology-based and *de novo* approaches. For the homology-based prediction, we used RepeatMasker version 4.0.6^19^ with the settings *“-nolow -no_is -norna -engine ncbi”* and RepeatProteinMask (a program within RepeatMasker package) with the settings *“-engine ncbi -noLowSimple -pvalue 0.0001”* to search TEs at the nucleotide and amino acid level based on known repeats (Fig. 3). RepeatMasker was applied for DNA-level identification using a custom library which combined the Repbase21.10 dataset^20^. At the protein level, RepeatProteinMask was used to perform RMBlast against the TE protein database. For *ab initio* prediction, RepeatModeler (v1.0.8)^21^ and LTR_FINDING (v1.06)^22^ were applied to construct the *de novo* repeat library. Contamination and multi-copy sequences in the library were removed and the remaining sequences were classified according to the BLAST result following alignment to the SwissProt database. Based on this library, we used RepeatMasker to mask the homologous TEs and classified them (Fig. 4). Overall, a total of 826 Mb of repetitive elements were identified in the spotted hyena, comprising 35.29% of the whole genome (Table 4).

**Table 4:** Transposable element content of the *Crotuta crotuta* genome assembly.

Type	Repbase TEs		TE proteins		*De novo*		Combined TEs	
	Length (Bp)	% in genome	Length (Bp)	% in genome	Length (Bp)	% in genome	Length (Bp)	% in genome
DNA	31,876,726	1.363	3,412,555	0.146	7,393,597	0.316	36,267,025	1.550
LINE	339,200,296	14.499	170,498,611	7.288	632,838,108	27.051	724,160,429	30.955
SINE	11,338,125	0.485	—	0.000	275,513	0.012	11,581,445	0.495
LTR	70,707,125	3.022	5,584,554	0.239	120,203,594	5.138	183,063,245	7.825
Other	115	0.000	—	0.000	—	0.000	115	0.000
Unknown	—	0.000	—	0.000	399,478	0.017	399,478	0.017
Total	452,356,734	19.336	179,484,993	7.672	696,609,269	29.777	825,501,231	35.287

Note: Repbase TEs: the result of RepeatMasker based on Repbase; TE proteins: the result of RepeatProteinMask based on Repbase; RepeatMasker: *de novo* finding repeats (Reaptmodeler and LTR_FINDING); Combined: the results obtained from combining the results using all the approaches.

### Protein-coding gene annotation

We used *ab initio* prediction and homolog-based approaches to annotate protein-coding genes as well splicing sites and alternative splicing isoforms. *Ab initio* prediction was performed on the repeat-masked genome using gene models from human, domestic dog, and domestic cat using AUGUSTUS (version 2.5.5)^23^, GENSCAN^24^, GlimmerHMM (version 3.0.4)^25^, and SNAP (version 2006-07-28)^26^, respectively. A total of 22,789 genes were identified by this method. Homologous proteins of, *Homo sapiens*, *Felis catus* and *Canis familiaris* (from the Ensembl 96 release) were mapped to the spotted hyena genome using tblastn (Blastall 2.2.26)^27^ with parameters “-e 1e-5”. The aligned sequences as well as their query proteins were then submitted to GeneWise (version 2.4.1)^28^ for searching an accurate spliced alignment. The final gene set (22,747) was collected by merging *ab initio* and homolog-based results using a customized pipeline (Table 5).

**Table 5:** General statistics of the number of protein-coding genes based on *ab initio* (*de novo*) and homology-based prediction methods.

Type		Gene number	Average transcript length (bp)	Average CDS length (bp)	Average exon per gene	Average exon length (bp)	Average intron length (bp)
Homolog	*Homo sapiens*	27757	19843.18	1226.43	6.87	178.41	3169.17
	*Canis familiaris*	60811	13539.62	876.21	4.92	178.24	3233.87
	*Felis catus*	203465	3981.86	349.47	2.39	146.33	2616.6
*De novo*	Augustus	19417	55148.7	1469.95	9.23	159.26	6522.36
	Genescan	43869	36636.74	1255.66	8.02	156.54	5039.23
	GlimmerHMM	7094	13731.07	1707.37	5.1	335.01	2935.17
	SNAP	69478	28690.76	685.71	5.74	119.5	5910.82
Final Gene set		22747	46986.43	1798.44	10.64	168.98	4686.16

### Gene function annotation

Gene functions were assigned according to the best match obtained by aligning translated gene coding sequences using BLASTP with parameters “-e 1e-5” to the SwissProt and TrEMBL databases (Uniprot release 2017-09). The motifs and domains of genes were determined by InterProScan (v5)^29^ against protein databases including ProDom^30^, PRINTS^31^, Pfam^32^, SMART^33^, PANTHER^34^ and PROSITE^35^. Gene Ontology IDs for each gene were obtained from the corresponding SwissProt and TrEMBL entries. All genes were aligned against KEGG proteins, and the pathway in which the gene might be involved was derived from the matched genes in the KEGG database^36^. In summary, 22,166 (97.45%) of the predicted protein-coding genes were successfully annotated by at least one of the six databases (Table 6).

**Table 6:** Number of genes with predicted homology or functional classification according to alignment to different protein databases.

	Number	Percent (%)
Total	22,747	100
InterPro	18,825	82.76
GO	9,052	39.79
NR	22,122	97.25
KEGG	18,778	82.55
Swissprot	21,251	93.42
TrEMBL	22,163	97.43
Annotated	22,166	97.45
Unannotated	581	2.55

### Gene family construction and phylogeny reconstruction

**Fig. 5:**
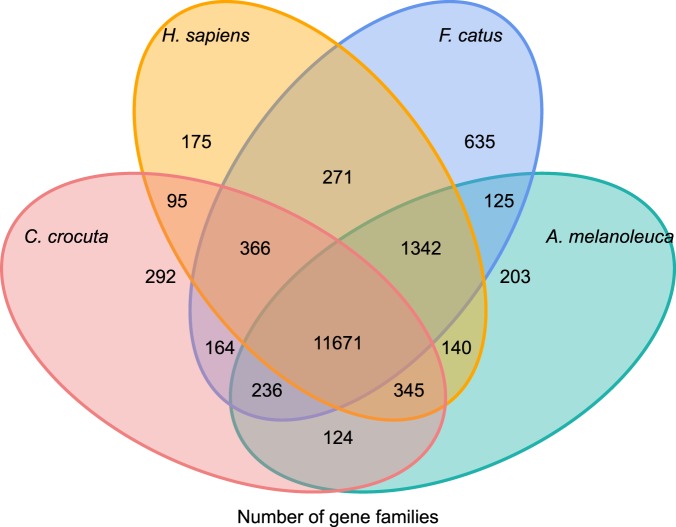
Venn diagram showing comparison of shared and unique protein-coding genes among spotted hyena, human, domestic cat and domestic dog based on orthology analysis.

To gain insight into the phylogenetic history and evolution of gene families of *Crocuta crocuta*, we clustered gene sequences of seven species (*Felis catus, Canis familiaris, Ailuropoda melanoleuca, Crocuta crocuta, Panthera pardus, Panthera leo, Panthera tigris altaica*) and *Homo sapiens* as the outgroup (Ensembl release-96, *Panthera leo* from unpublished data) into gene families using orthoMCL (v2.0.9)^37^. The protein-coding genes for the eight species were retrieved by selecting the longest transcript isoform for each gene for downstream pairwise assignment (graph building). We performed an all-against-all BLASTP search on the protein sequences of all the reference species, with an E-value cut-off of 1e-5. Gene family construction employed the MCL algorithm^38^ with the inflation parameter of ‘1.5’. A total of 16,271 gene families of *C. crocuta*, *H. sapiens*, *F. catus*, *A. melanoleuca* were clustered. There were 11,671 gene families shared by these four species, while 292 gene families containing 1,446 genes were specific to *C. Crocuta* (Fig. 5). Noticeably, the gene families *C. crocuta* and *F. catus* shared were less than *C. crocuta* and *H. sapiens* shared, which could result from that *H. sapiens* had a more complete genome and annotation.

**Fig. 6:**
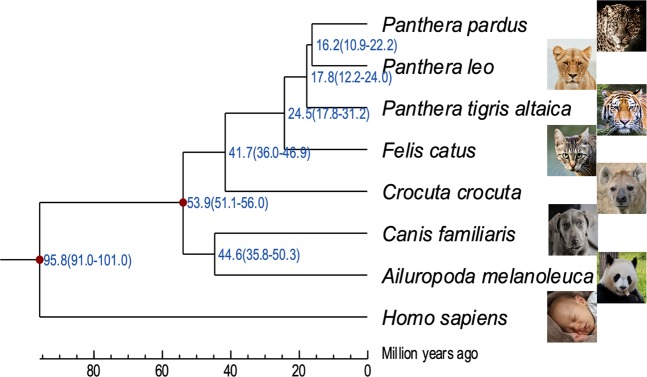
Phylogenetic tree of *C. crocuta* and seven other species constructed by the maximum likelihood method based on 6,601 single-copy orthologues. The divergence time was estimated using the two calibration priors derived from the Time Tree database (http://www.timetree.org), which are marked by a red rhombus. All estimated divergence times are shown with 95% confidence intervals in brackets.

We identified 6,601 single-copy orthologous genes to reconstruct the phylogenetic tree of the eight species. Multiple sequence alignments of amino acid sequences for each gene were generated using MUSCLE (version 3.8.31)^39^, and trimmed using Gblocks (0.91b)^40^, achieving well-aligned regions with the parameters *“-t* = *p -b3* = *8 -b4* = *10 -b5* = *n -e* = *-st*”. We performed phylogenetic analysis using the maximum-likelihood method as implemented in PhyML (v3.0)^41^, using the JTT + G + I model for amino acid substitution (Fig. 6). The root of the tree was determined by minimizing the height of the whole tree via Treebest (v1.9.2; http://treesoft.sourceforge.net/treebest.shtml). Finally, we estimated the divergence time among the eight lineages using MCMCTree from the PAML version 4.4 software package^42^. Two priors based on the fossil record were used to calibrate the substitution rate, including *Boreoeutheria* (91-102 MYA) and *Carnivora* (52-57 MYA)^43^. Consistent with previous studies, the spotted hyena groups with the four species included from the Felidae in a clade defining the suborder Feliformia, which diverged from the Caniformia (represented by the domestic dog and giant panda) 53.9 Mya^44^.

## Data Records

Raw reads from Illumina sequencing are deposited in the NCBI Sequence Read Archive (SRA) database with accession numbers SRP215800^45^, and Bioproject accession PRJNA554753 and are also deposited in CNGB Nucleotide Sequence Archive (CNSA) database with accession number CNR0105011-CNR0105023^46–58^ and Bioproject accession CNP0000511. The genome assembly of *C. crocuta* generated in this study was deposited in NCBI Assembly under the accession number GCA_008692635.1^59^ and in CNSA with the accession number CNA0003520^60^. Copies of all of annotation outputs including genes, functional assignments, and copies of the gene families and its statistics, and final tree in newick format for 8 species are deposited in figshare database^61^.

## Technical Validation

### DNA quality control

Quantification of the DNA sample using both NanoDrop and a DNA fluorometer were performed before library construction (see method). DNA sample was also identified by DNA barcode of Cytb gene to avoid a mislabeling.

### Comparison for genome assembly of striped hyena

**Fig. 7:**
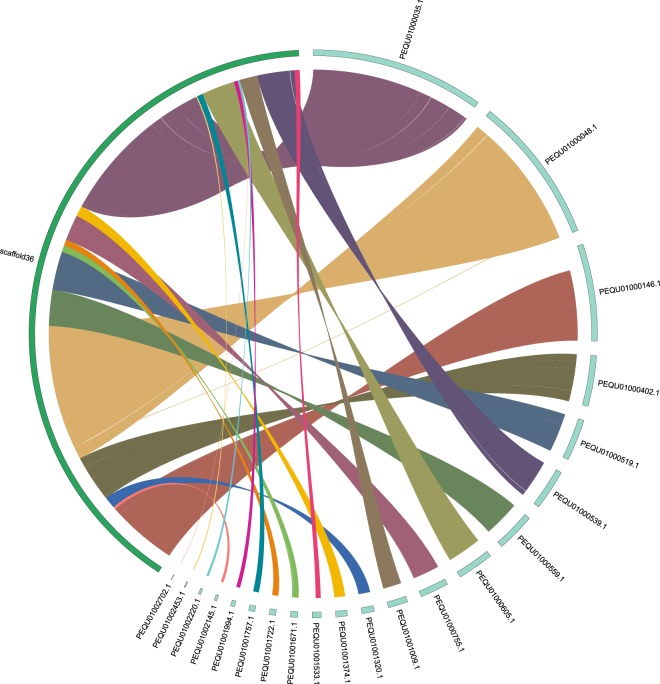
A case of syntenic relationship of spotted hyena genome with striped hyena genome assembly (GCA_003009895.1). The right dark green scaffold belongs to spotted hyena, whereas the left light green scaffolds belong to striped hyena. Links connect the location of homeologs blocks between two genome assemblies, based on the comparison of sequence information using LASTZ (version 1.02.00, http://www.bx.psu.edu/~rsharris/lastz/) under the default settings. The scaffolds of length >1 Mb and links (syntenic block) of length >100 kb are showed on figure.

The previous released genome assemblies of striped hyena (*Hyaena hyaena*) were relatively fragmented and had scaffold N50 of 66,490 bp and 2,001,327 bp, respectively, which are significantly shorter than the presented spotted hyena genome assembly has scaffold N50 of 7,168,038 bp (Table 7). We chose the better assembled genome of striped hyena (GCA_003009895.1) to conduct a synteny analysis with spotted hyena. The synteny analysis revealed that the two genome assemblies had overall a high ratio of syntenic region, with 96% for spotted hyena and 90.5% for striped hyena can be mapped to each other. However, the spotted hyena genome assembly has less breakpoints and has more contiguous than striped hyena genome (Fig. 7). On average, each spotted hyena scaffold can be mapped to 1.07 scaffolds of striped hyena. Overall, the spotted genome assembly has much higher quality and can be valuable for future comparative genomics study for hyena and other mammals.

**Table 7:** Overall statistic of syntenic analysis between spotted hyena and striped hyena.

	Spotted hyena	Striped hyena GCA_004023945.1	Striped hyena GCA_003009895.1
Total length of genome assembly (bp)	2,355,303,269	2,445,474,026	2,374,716,107
Genome coverage (X)	95.2	31.3	56
Contig N50 (bp)	21,301	51,677	311,202
Scaffold N50 (bp)	7,168,038	66,490	2,001,327
GC content	41.10%	41.64%	41.27%
Number of Scaffolds	171,240	350,223	5,760
BUSCO result	C:95.5%[S:95.0%,D:0.5%],F:2.5%,M:2.0%,n:4104	C:74.7%[S:73.7%,D:1.0%],F:16.2%,M:9.1%,n:4104	C:95.6%[S:94.9%,D:0.7%],F:2.4%,M:2.0%,n:4104

## Data Availability

The bioinformatic tools used in this work, including versions, settings and parameters, have been described in the Methods section. Default parameters were applied if no parameters were mentioned for a tool. The scripts used in generating the orthoMCL results and preparing input sequences for PhyML were deployed on the Github repository (https://github.com/comery/For_soptted_hyena_genome).
